# Multivariate pattern dependence

**DOI:** 10.1371/journal.pcbi.1005799

**Published:** 2017-11-20

**Authors:** Stefano Anzellotti, Alfonso Caramazza, Rebecca Saxe

**Affiliations:** 1 Brain and Cognitive Sciences Department, MIT, Cambridge, Massachusetts, United States of America; 2 Department of Psychology, Harvard University, Cambridge, Massachusetts, United States of America; Oxford University, UNITED KINGDOM

## Abstract

When we perform a cognitive task, multiple brain regions are engaged. Understanding how these regions interact is a fundamental step to uncover the neural bases of behavior. Most research on the interactions between brain regions has focused on the univariate responses in the regions. However, fine grained patterns of response encode important information, as shown by multivariate pattern analysis. In the present article, we introduce and apply multivariate pattern dependence (MVPD): a technique to study the statistical dependence between brain regions in humans in terms of the multivariate relations between their patterns of responses. MVPD characterizes the responses in each brain region as trajectories in region-specific multidimensional spaces, and models the multivariate relationship between these trajectories. We applied MVPD to the posterior superior temporal sulcus (pSTS) and to the fusiform face area (FFA), using a searchlight approach to reveal interactions between these seed regions and the rest of the brain. Across two different experiments, MVPD identified significant statistical dependence not detected by standard functional connectivity. Additionally, MVPD outperformed univariate connectivity in its ability to explain independent variance in the responses of individual voxels. In the end, MVPD uncovered different connectivity profiles associated with different representational subspaces of FFA: the first principal component of FFA shows differential connectivity with occipital and parietal regions implicated in the processing of low-level properties of faces, while the second and third components show differential connectivity with anterior temporal regions implicated in the processing of invariant representations of face identity.

## Author summary

Human behavior is supported by systems of brain regions that exchange information to complete a task. This exchange of information between brain regions leads to statistical relationships between their responses over time. Most likely, these relationships do not link only the mean responses in two brain regions, but also their finer spatial patterns. Analyzing finer response patterns has been a key advance in the study of responses within individual regions, and can be leveraged to study between-region interactions. To capture the overall statistical relationship between two brain regions, we need to describe each region’s responses with respect to dimensions that best account for the variation in that region over time. These dimensions can be different from region to region. We introduce an approach in which each region’s responses are characterized in terms of region-specific dimensions that best account for its responses, and the relationships between regions are modeled with multivariate linear models. We demonstrate that this approach provides a better account of the data as compared to standard functional connectivity in two different experiments, and we use it to discover multiple dimensions within the fusiform face area that have different connectivity profiles with the rest of the brain.

This is a *PLOS Computational Biology* Methods paper.

## Introduction

Cognitive tasks recruit multiple brain regions [1–4]. How do these regions work together to generate behavior? A variety of methods have been developed to study connectivity both in terms of the anatomical structure of the brain [5], and of the relations between timecourses of responses during rest [6] and during specific experimental tasks [7–11]. Functional Magnetic Resonance Imaging (fMRI) has proven to be a valuable instrument in this enterprise, offering noninvasive recording with good spatial resolution and whole-brain coverage.

In parallel to this literature, multivariate pattern analysis (MVPA; [12]) has drastically increased the potential of fMRI for the investigation of representational content, making it possible to detect information at a level of specificity that was unthinkable with previous univariate analyses [13–17]. Despite the success of MVPA, relatively few attempts have been made to transport the potential of multivariate analyses to the domain of dynamics and connectivity.

A recent study [18] used trial-by-trial classification accuracy of color and shape in area V4 and in the lateral occipital complex (LOC) to predict trial-by-trial accuracy of object classification in the anterior temporal lobe (ATL). Earlier work by the same group [19] used a continuous measure of classification based on correlations, offering a richer description of each brain region’s patterns. These studies are important steps towards exploiting the wealth of information encoded in patterns of BOLD response to study connectivity, but they both characterize the information encoded in a brain region using a single measure (a given classification), rather than in terms of values along multiple dimensions.

An additional property of both these methods [18, 19] is that they use classification along experimenter defined categories. This approach can be useful to probe a specific hypothesis about a given classification. However, it might disregard other information encoded by the regions studied which is orthogonal to the categories chosen by the experimenter. As a consequence, the results depend on the experimenter’s choice of the categories, and on how well the chosen categories capture the functional role of the regions studied.

Multivariate pattern dependence (MVPD) is a novel method to investigate the ‘connectivity’ between brain regions in terms of multivariate spatial patterns of responses. In keeping with the statistical literature [20], we will replace the term ‘connectivity’ with the term ‘statistical dependence’, which we consider more accurate. MVPD is composed of three main stages. In the first stage, the representational space in each brain region is modeled extracting a set of data-driven dimensions (rather than chosen by the experimenter), that correspond to spatial response patterns that ‘best’ characterize that region’s responses over time. In the second stage, the multivariate timecourses of responses in each region are reparametrized as trajectories in the representational spaces defined by these dimensions. In the third stage, the multivariate relations between the trajectories in the representational spaces of different regions are modelled. In a procedure analogous to MVPA, independent data are used to train and test the models. The dimensions and the parameters modelling the relationship between two regions are estimated with all runs but one, and then used to model the relation between those regions in the remaining run.

We demonstrate the potential of MVPD in two different experiments, analyzing the statistical dependence between the posterior superior temporal sulcus (pSTS) during the recognition of faces and voices, and of the fusiform face area (FFA) during the recognition of faces. In both experiments, MVPD identified dependencies between regions not detected by standard functional connectivity, and explained more variance in individual voxels responses than univariate methods. In the end, MVPD revealed different connectivity profiles associated with different dimensions of FFA’s responses.

## Materials and methods

### Ethics statement

The volunteers’ consent was obtained according to the Declaration of Helsinki (BMJ, 1991, pp. 302, 1194). The project was approved by the Human Subjects Committees at the University of Trento and Harvard University.

### Experiment 1

#### Participants

Eleven volunteers (6 female; age range: 19-32, mean = 24) took part in the experiment.

#### Stimuli

The faces and voices of three famous Italian politicians (Matteo Renzi, Pierluigi Bersani, and Silvio Berlusconi) were used as stimuli. Two grayscale images of each face were selected and cropped to an oval, and equated in luminance and contrast. Two audio clips were selected for each of the three politicians: one in which they said “Italia” (“Italy”) and one in which they said “governo” (“government”). The audio stimuli were further equated in loudness.

#### Experimental design

Inside the scanner, participants completed two localizer runs (a face localizer and a voice localizer) and five experimental runs. Before entering the scanner, participants were instructed to consider a given individual (e.g., Matteo Renzi) as the target. Participants were instructed to press a button with the index finger of the right hand when the target was presented, and a button with the middle finger of the right hand when a distractor was presented, irrespectively of stimulus modality. In the face localizer, participants were shown 16 seconds blocks of faces and houses, and performed a 1-back task reporting whether a stimulus was identical to the one that had been presented in the previous trial. In the voice localizer, participants heard 16 seconds blocks of voices and tool sounds, and performed an analogous 1-back task. In each experimental run, each ‘distractor’ face and each ‘distractor’ voice was presented 16 times, while the target face and target voice were presented for 8 trials. Given that there are two ‘distractor’ identities and one ‘target’ identity, this implies that the target identity was presented on 20% of the trials. Stimuli were presented for 500ms and were followed by a 3500ms fixation. The order of the trials was optimized to maximize efficiency using Optseq 2 (http://surfer.nmr.mgh.harvard.edu/optseq/). Data from Experiment 1 has been previously used to investigate representations of person identity [21].

### Experiment 2

#### Participants

A total of ten volunteers (N = 3 female, age range 18-50, mean 27.1) participated in the experiment. Data from one participant were discarded from the analysis because of poor performance during a behavioral training session administered on the day before the scanning.

#### Stimuli

Computer generated 3D models (using DAZ-3D) of 5 face identities were used to generate images at 5 different orientations for each identity (S1 Fig). Stimuli were presented with Psychtoolbox [22, 23] running on MATLAB, with the add-on ASF [24], using an Epson EMP 9000 projector. Images were projected on a frosted screen at the top of the bore, viewed through a mirror attached to the head coil.

#### Experimental design

One of the five face identities was designated as the ‘target’, and participants were instructed to respond with the index finger of the right hand to the target face and with the middle finger to the other ‘distractor’ faces. Each trial consisted of the presentation of a face image (500ms) followed by a fixation cross (1500ms). The experiment was composed of three 12-minute runs, each consisting of approximately 320 trials. The order of presentation of the stimuli was generated with optseq2 (http://surfer.nmr.mgh.harvard.edu/optseq/). A 6 minutes block-design functional localizer was administered at the beginning of the fMRI session. Participants observed 16 second long blocks comprising 8 images of faces, 8 images of houses, or 8 scrambled images, and performed a 1-back task in which they had to detect repetitions of identical stimuli. None of the faces shown in the localizer were presented during the other parts of the experiment. Data from Experiment 2 has been previously used to investigate representations of face identity [15].

### Data acquisition

The data were collected on a Bruker BioSpin MedSpec 4T at the Center for Mind/Brain Sciences (CIMeC) of the University of Trento using a USA Instruments eight-channel phased-array head coil. Before collecting functional data, a high-resolution (1 × 1 × 1 *mm*^3^) T1-weighted MPRAGE sequence was performed (sagittal slice orientation, centric phase encoding, image matrix = 256 × 224 [Read × Phase], field of view = 256 × 224 *mm*^2^ [Read × Phase], 176 partitions with 1 *mm* thickness, GRAPPA acquisition with acceleration factor = 2, duration = 5.36 minutes, repetition time = 2700, echo time = 4.18, *TI* = 1020 *msec*, 7° flip angle). Functional data were collected using an echo-planar 2D imaging sequence with phase oversampling (image matrix = 70 × 64, repetition time = 2000 *msec*, echo time = 21 *msec*, flip angle = 76°, slice thickness = 2 *mm*, gap = 0.30 *mm*, with 3 × 3 *mm* in plane resolution). Over three runs, 1095 volumes of 43 slices were acquired in the axial plane aligned along the long axis of the temporal lobe.

### Preprocessing and de-noising

Data were preprocessed with SPM12 (http://www.fil.ion.ucl.ac.uk/spm/software/spm8/) and regions of interest were generated with MARSBAR [25] running on MATLAB 2010a. Subsequent analyses were performed with custom MATLAB software. The first 4 volumes of each run were discarded and all images were corrected for head movement. Slice-acquisition delays were corrected using the middle slice as reference. Images were normalized to the standard SPM12 EPI template and resampled to a 2 *mm* isotropic voxel size. The BOLD signal was high pass filtered at 128*s* and prewhitened using an autoregressive model AR(1). Outliers were identified with the artifact removal tool (ART), using both the global signal and composite motion. Datapoints exceeding experimenter-defined thresholds were removed from the analysis. An additional noise-removal step was performed with CompCorr [26]. In each individual participant, a control region was defined combining the white matter and cerebrospinal fluid masks obtained with SPM segmentation, and five principal components were extracted. Since the control region does not contain gray matter, its responses are thought to reflect noise. For each run, the timecourses of the components extracted from the control region were regressed out from the timecourses of every voxel in gray matter. For both experiments, the global signal and six motion regressors generated by SPM during motion correction were also included as regressors of no interest. For the FFA seed, data were analyzed both with and without these additional regressors, and results are reported for both analyses.

### ROI definition

For experiment 1, we defined a seed region of interest in the right pSTS using the independent functional localizer. Data were modeled with a standard GLM using SPM12, and the seed ROI was defined in each individual participant as a 6mm radius sphere centered in the pSTS peak for the faces vs houses contrast (mean MNI coordinates: 54,-54,13).

For experiment 2, we defined a seed region of interest in the right FFA using the independent functional localizer. Data were modeled with a standard GLM using SPM12, and the seed ROI was defined in each individual participant as a 6mm radius sphere centered in the FFA peak for the faces vs houses contrast (mean MNI coordinates: 40,-48,-20).

### Searchlight

We defined a gray matter mask by smoothing (with a 6mm FWHM gaussian kernel) and averaging the gray matter probabilistic maps obtained during segmentation. The average maps were then thresholded obtaining approximately 130000 gray matter voxels (127821). For each voxel in the gray matter mask, we defined a 6mm radius sphere centered in that voxel, and calculated the statistical dependence between the responses in the seed region and the responses in the sphere. Spheres contained 123 voxels. Spheres at the edge of the brain were restricted to the voxels within the gray matter mask.

### Standard functional connectivity

Functional connectivity was calculated low-pass filtering at 0.1*Hz* the mean response in the seed region and the mean response in the searchlight spheres, and calculating Pearson’s correlation between the low-pass filtered responses in the seed and each sphere, thus obtaining a whole-brain functional connectivity map. Statistical significance across participants was assessed with statistical nonparametric mapping [27] using the SnPM extension for SPM (http://warwick.ac.uk/snpm).

### MVPD: Modeling representational spaces

Let us consider the multivariate timecourses in the seed region: *Y*_1_, …, *Y*_*m*_ and in a sphere: *X*_1_, …, *X*_*m*_, for experimental runs from 1 to *m*. Each multivariate timecourse *Y*_*i*_ is a matrix of size *T*_*i*_ × *n*_*y*_, where *n*_*y*_ is the number of voxels in the seed region and *T*_*i*_ is the number of timepoints in run *i*. Analogously, each multivariate timecourse *X*_*i*_ is a matrix of size *T*_*i*_ × *n*_*x*_, where *n*_*x*_ is the number of voxels in the sphere. Data analysis followed a leave-one-run-out procedure: for each choice of an experimental run *i*, data in the remaining runs were concatenated, obtaining

$$Y_{train}=(Y_{1},\ldots ,Y_{i-1},Y_{i+1},\ldots ,Y_{m});$$

$$X_{train}=(X_{1},\ldots ,X_{i-1},X_{i+1},\ldots ,X_{m}).$$

Principal component analysis (PCA) was applied to *Y*_*train*_, and *X*_*train*_:

$$Y_{train}=U_{Y}S_{Y}V_{Y}^{T}$$

$$X_{train}=U_{X}S_{X}V_{X}^{T}$$

Dimensionality reduction was implemented projecting *Y*_*train*_ and *X*_*train*_ on lower dimensional subspaces spanned by the first *k*_*Y*_ and *k*_*X*_ principal components respectively:

$${\tilde{Y}}_{train}=Y_{train}V_{Y}^{\left[1,\ldots ,k_{Y}\right]}$$

$${\tilde{X}}_{train}=X_{train}V_{X}^{\left[1,\ldots ,k_{X}\right]}$$

where $V_{T}^{\left[1,\ldots ,k_{T}\right]}$ is the matrix formed by the first *k*_*Y*_ columns of *V*_*Y*_ and $V_{X}^{\left[1,\ldots ,k_{X}\right]}$ is the matrix formed by the first *k*_*X*_ columns of *V*_*X*_. In the first analysis, the number of components *k*_*Y*_ and *k*_*X*_ was chosen for each sphere and iteration using the Bayesian Information Criterion (BIC). In the second analysis, the incremental contribution of each component was tested by comparing the results obtained choosing 1, 2 and 3 components. We can take a moment to reflect on the interpretation of the procedure we just completed. For each region, each dimension obtained with PCA is a linear combination of the voxels in the region, whose weights define a multivariate pattern of response over voxels. Considering as an example the seed region, the loadings of a dimension *j* are encoded in the *j*-th column of ${\tilde{Y}}_{train}$, and represent the intensity with which the multivariate pattern corresponding to dimension *j* is activated over time.

### MVPD: Modeling statistical dependence

The mapping *f* from the dimensionality-reduced timecourses in the sphere ${\tilde{X}}_{train}$ to the dimensionality-reduced timecourses in the seed ${\tilde{Y}}_{train}$ was modeled with multiple linear regression 1:

$$\begin{array}{c} {\tilde{Y}}_{train}=B_{train}{\tilde{X}}_{train}+E_{train} \end{array}$$ (1)

the model parameters were estimated using ordinary least squares (OLS).

### MVPD: Predicting multivariate timecourses

After having estimated parameters *B*_*train*_, predictions for the multivariate responses in the left out run *i* were generated by 1) projecting the sphere data in run *i* on the sphere dimensions estimated with the other runs, and 2) multiplying them by the parameters estimated using data from the other runs. More formally, for each run *i*, we generated dimensionality reduced responses in the sphere:

$${\tilde{X}}_{test}=X_{test}V_{X}^{\left[1,\ldots ,k_{X}\right]},$$

where *V*_*X*_ was calculated using the training data. Then, we calculated the predicted responses in the seed region in run *i*:

$${\hat{Y}}_{test}=B_{train}{\tilde{X}}_{test}$$

using the parameters *B*_*train*_ independently estimated with the training runs.

**Fig 1:**
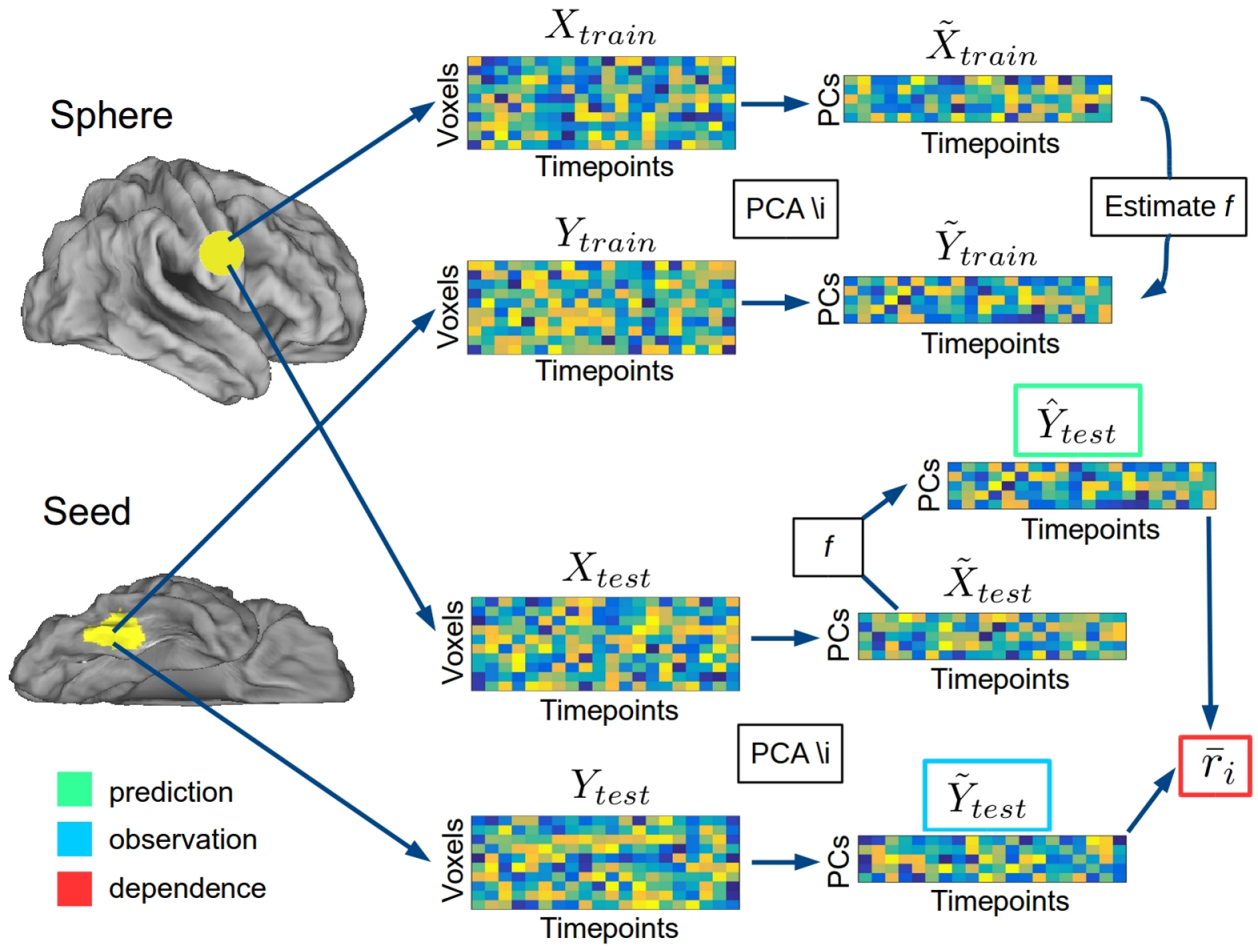
Analysis pipeline.

In keeping with the use of correlation in standard functional connectivity, we calculated the correlation between the predicted and observed timecourses in each dimension in the seed region. First, we projected the observed voxelwise timecourses in the seed region onto the lower dimensional subspace using $V_{Y}^{\left[1,\ldots ,k_{Y}\right]}$:

$${\tilde{Y}}_{test}=Y_{test}V_{Y}^{\left[1,\ldots ,k_{Y}\right]},$$ (2)

where *V*_*Y*_ was calculated using the training data. Then, we computed

$$r_{j}=\text{corr}\left({\hat{Y}}_{test}^{j},{\tilde{Y}}_{test}^{j}\right)$$

for each dimension *j* = 1, …, *k*_*Y*_ of the seed region’s subspace. In the end, we generated a single summary measure $\bar{r}$, computing the average of the values *r*_*j*_ weighted by the proportion of variance explained by the corresponding dimensions *j*:

$$w_{j}\;=\frac{S_{Y}(j,j)}{\sum_{l=1}^{k_{Y}}S_{Y}(l,l)},$$

$${\bar{r}}_{i}=\sum_{j=1}^{k_{Y}}w_{j}r_{j}$$

(see the relationship between the eigenvalues along the diagonal of *S* and variance explain in PCA). This procedure is motivated by the observation that if a dimension explains more overall variance in the total multivariate response, then explaining variability in that dimension should be weighted more. See Fig 1 for an outline of the method. The values ${\bar{r}}_{i}$ obtained for the different runs *i* = 1, …, *m* were averaged yielding $\bar{r}$. This procedure was repeated for each searchlight sphere, obtaining a whole brain map of $\bar{r}$ values for each participant. The significance of $\bar{r}$ was tested across participants with statistical nonparametric mapping [27] using the SnPM extension for SPM (http://warwick.ac.uk/snpm).

### Voxelwise variance explained

The value $\bar{r}$ is a convenient measure of statistical dependence: it reflects how well the prediction generated by MVPD correlates with the observed data. However, in this measure, the target of the prediction is the multivariate timecourse ${\tilde{Y}}_{test}$. Instead, ‘standard’ univariate connectivity based on the mean timecourse aims to predict a different target: mean(*Y*_*test*_, 2). This is important because the proportion of variance explained (cross-validated R-squared) is given by the amount of variance explained divided by the total variance of the target of the prediction. Univariate connectivity and MVPD could explain the same amount of absolute variance, but still have different proportions of variance explained, because the total variances of the targets of the prediction differ. One way to think about this is that mean-based univariate connectivity ‘gives up’ on predicting variability orthogonal to the mean: if the mean response is predicted perfectly, then the proportion of variance explained will be 100%. In contrast, if MVPD tries to predict the mean as well as other dimensions, it could predict the mean perfectly like univariate connectivity, and still its proportion of variance explained could be less than 100%, because of residuals in the other dimensions. To compare the cross-validated R-squared of univariate connectivity and of MVPD, therefore, we need a measure of their ability to predict a common target. For this reason, for each searchlight sphere we calculated the cross-validated R-squared of mean-based univariate connectivity and of MVPD in the timecourses of individual voxels in the seed region. Predicting the timecourses of all voxels in the seed region is a common target for both univariate connectivity and MVPD, and therefore it makes the cross-validated R-squared of the two methods comparable. To calculate the cross-validated R-squared for both methods, we needed to use a variant of functional connectivity that can perform leave-one-out predictions. The variance explained in functional connectivity is *r*^2^, and it is equal to the variance explained by a linear regression estimated and tested in the same data. We used linear regression estimated in all runs minus one, and tested the variance explained in the left-out run, thus obtaining a leave-one-out variant of mean-based univariate functional connectivity (that uses the same data-split used in MVPD). The linear regression yielded a prediction of the mean response in the seed region. Each voxel’s response was then predicted with the predicted mean response in the seed region. For MVPD, we predicted each voxel’s response projecting the multivariate prediction ${\hat{Y}}_{test}$ from its low-dimensional subspace of principal components to voxel space, using the matrix $V_{Y}^{\left[1,\ldots ,k_{Y}\right]}$. Each voxel’s response was reconstructed as the sum of the dimensions’ loadings on the voxels weigthed by the dimensions’ loadings at each timepoint. It can be helpful here to note that this is equivalent to the product

$${\overset{˘}{Y}}_{test}={\hat{Y}}_{test}V_{Y}^{[1,\ldots ,k_{Y}]T},$$

where ${\overset{˘}{Y}}_{test}$ is the voxel-wise prediction (see 2 and consider that $(V_{Y}^{\left[1,\ldots ,k_{Y}\right]}{)}^{T}=(V_{Y}^{\left[1,\ldots ,k_{Y}\right]}{)}^{-1}$). In the case of the mean-based univariate functional connectivity, the voxelwise prediction can be written as

$${\overset{˘}{Y}}_{test}={\hat{Y}}_{test}1^{T},$$

where ${\hat{Y}}_{test}$ is the predicted mean response in the seed region and **1** is a *n*_*Y*_ × *T*_*i*_ vector of ones, making explicit the common form of the prediction for MVPD and for mean-based univariate connectivity: in the latter the mean is treated as a single dimension with equal loadings for each voxel.

For each voxel *j* in the seed region, variance explained was calculated as

$$v\left(j\right)=1-\frac{SS(Y_{test}\left(:,j\right)-{\overset{˘}{Y}}_{test}\left(:,j\right))}{SS(Y_{test}\left(:,j\right))}$$

where ${\overset{˘}{Y}}_{}$ are the predicted voxelwise timecourses, and the values *v*(*j*) were averaged to obtain a single measure

$$\bar{v}=\frac{\sum_{j=1}^{n_{Y}}v\left(j\right)}{n_{Y}}$$

for each searchlight sphere.

## Results

**Fig 2:**
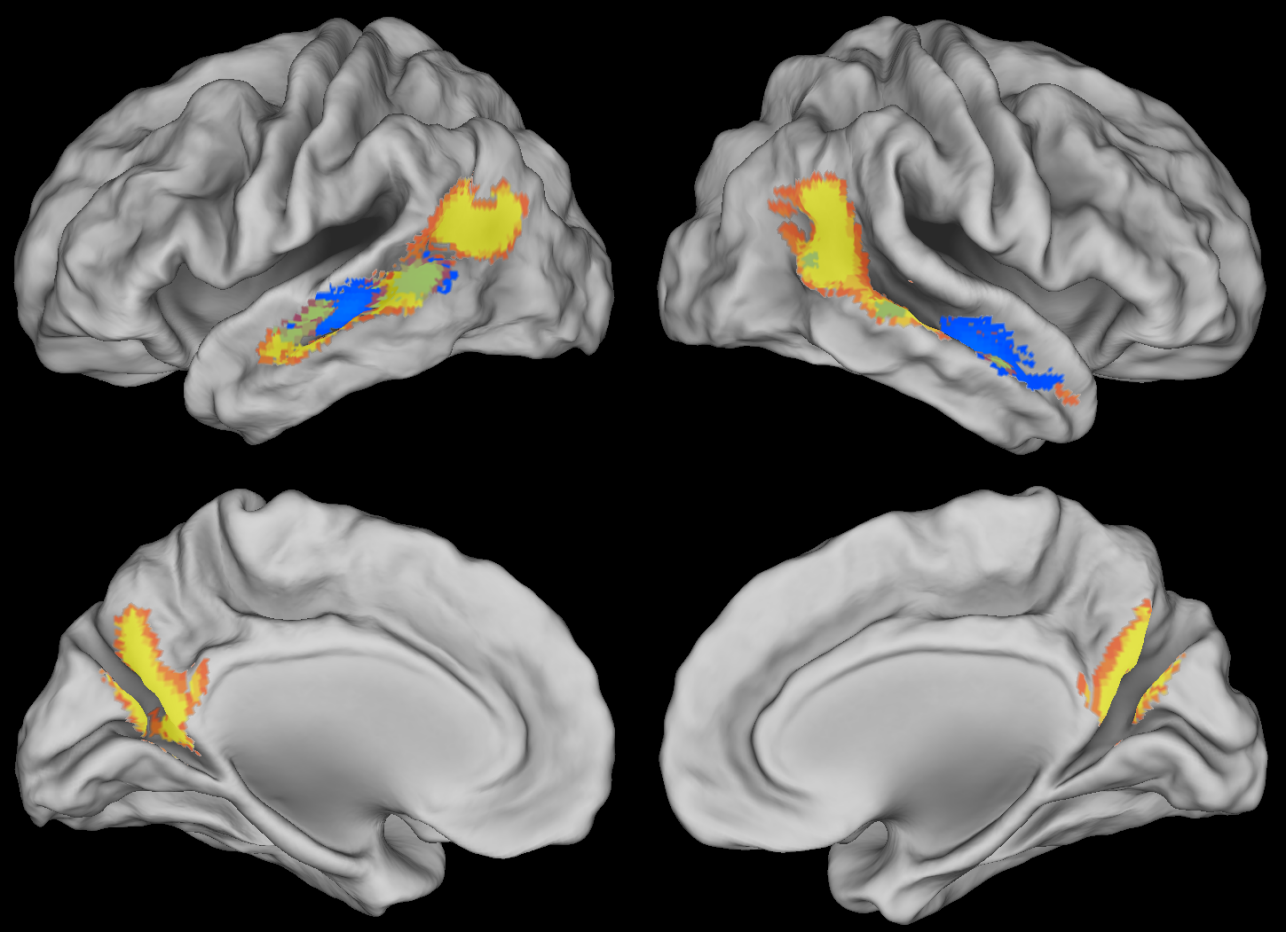
Brain regions showing statistical dependence with the right pSTS as identified by standard functional connectivity (blue) and multivariate pattern dependence (MVPD, yellow) at a voxelwise FWE-corrected threshold *p* < 0.05. MVPD additionally identified statistical dependence with the posterior cingulate, and with larger portions of posterior STS bilaterally.

In Experiment 1, standard functional connectivity identified statistical dependence between the right pSTS and more anterior regions of right STS (peak MNI: 54 -9 -15) and with the left STS (peak MNI: -52 -27 -6) (Fig 2, S1 Table). MVPD, but not standard functional connectivity, identified statistical dependence with the posterior cingulate (peak MNI: 0 -71 34), and with larger portions of posterior STS bilaterally (Fig 2, S2 Table).

**Fig 3:**
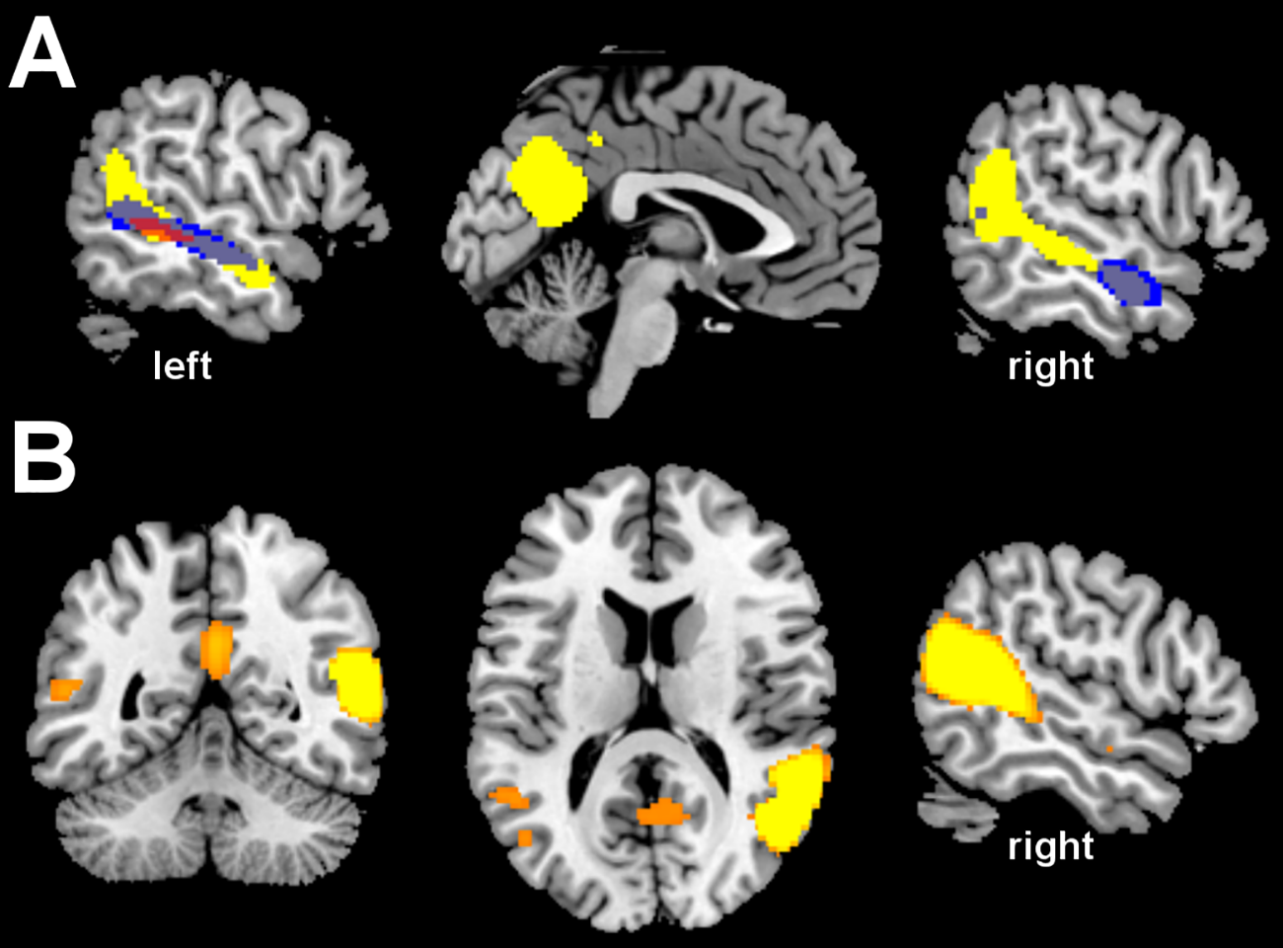
A) Comparison between statistical dependence measured with standard functional connectivity (‘univariate dependence’, blue), univariate dependence with leave-one-out predictions (red), and multivariate dependence with leave-one-out predictions (MVPD, yellow) at a voxelwise FWE-corrected threshold *p* < 0.05. Predicting independent data is a more stringent test of dependence: univariate dependence with leave-one-out predictions individuates fewer significant voxels than standard functional connectivity. Despite the stringent criterion imposed by independent predictions, MVPD with leave-one-out predictions outperforms both univariate dependence methods, identifying significant statistical dependence with regions of posterior cingulate and a broader extent of the superior temporal sulcus. B) Cross-validated R-squared with MVPD, thresholded at 20%.

To evaluate the separate effects of predicting independent data with a leave-one-out approach and of transitioning from univariate to multivariate statistical dependence, we additionally measured univariate statistical dependence with a leave-one-out procedure. As anticipated, predicting independent data reduced the number of significant voxels for univariate dependence (or ‘connectivity’) as compared to standard functional connectivity (Fig 3A), in line with the expectation that predicting independent data is a more stringent test. MVPD, despite predicting independent data, outperformed both variants of univariate dependence (Fig 3A). As a further comparison between univariate dependence and MVPD, we calculated the proportion of variance explained by each model in independent data. Univariate dependence did not explain more than 5% of the variance in any brain region, while MVPD explained more than 20% of the variance in several regions, including the STS bilaterally and posterior cingulate (Fig 3B).

**Fig 4:**
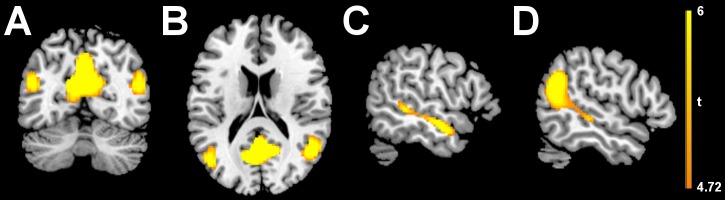
MVPD calculated after removing the univariate signal, in coronal (A), axial (B), and sagittal left (C) and right (D) views. In this analysis, the average timecourse in the seed and each sphere is zero, and only the patterns of responses are left. As a consequence, this analysis is fully complementary to standard functional connectivity. Even after removing the univariate signal, MVPD detected significant statistical dependence between the right pSTS and posterior cingulate as well as the left STS.

As an additional test of the potential of MVPD, we analyzed multivariate dependence between the pSTS seed and the rest of the brain after subtracting the univariate signal (Fig 4). By doing so, we obtained an analysis procedure which is entirely complementary to standard functional connectivity, which relies entirely on the univariate signal. Even after removing the univariate signal, MVPD detected significant statistical dependence between the right pSTS and posterior cingulate (peak MNI: 0 -63 28) as well as the left STS (peak MNI: -58 -10 -13).

**Fig 5:**
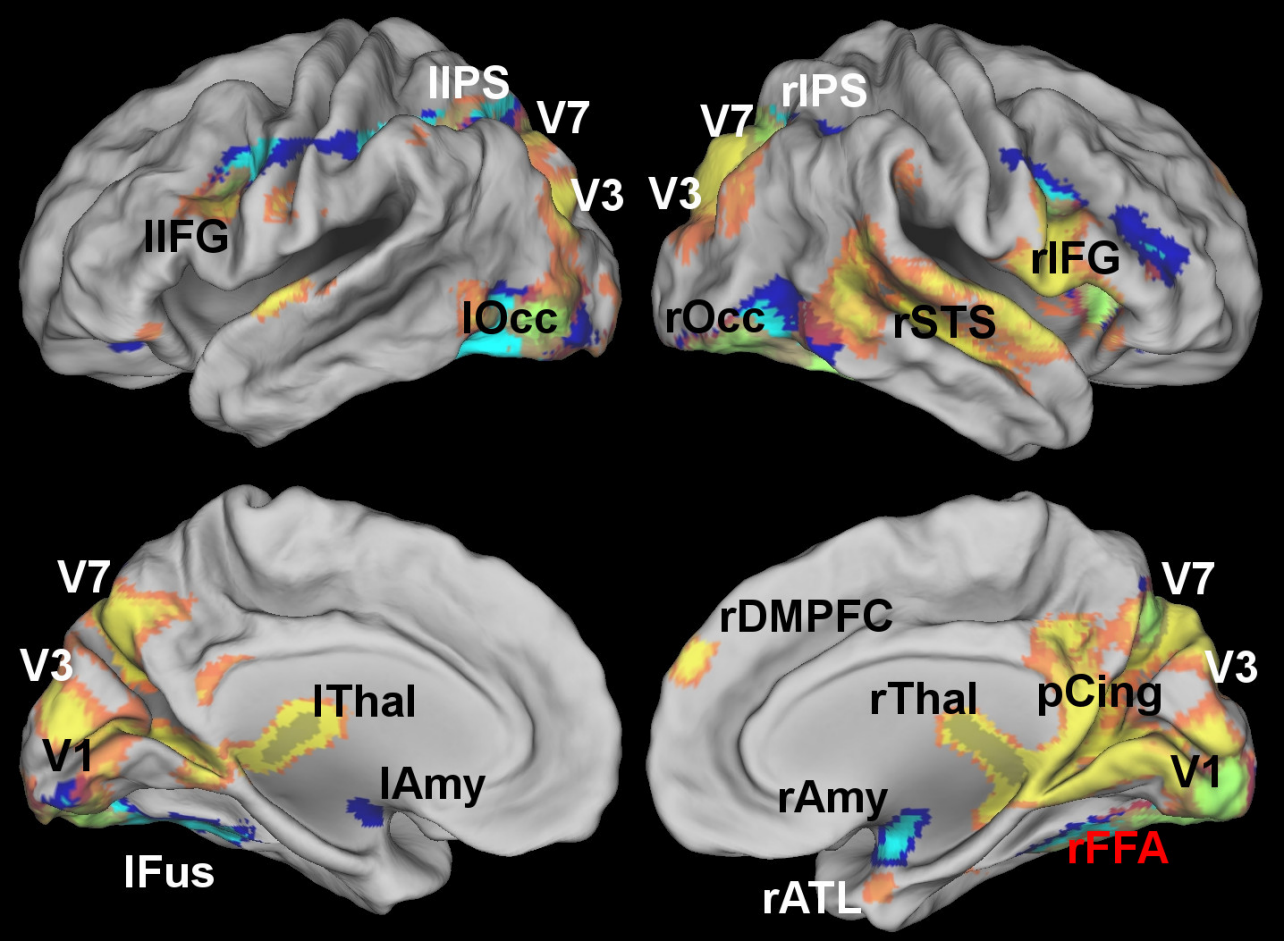
Brain regions showing statistical dependence with the FFA as identified by standard functional connectivity (blue) and multivariate pattern connectivity (MVPD, yellow) at a voxelwise FWE-corrected threshold *p* < 0.05. MVPD, but not standard functional connectivity, identified statistical dependence with regions of posterior cingulate, the right superior temporal sulcus, the right anterior temporal lobe, the right DMPFC and regions of the dorsal visual stream. Standard functional connectivity identified statistical dependence with the amygdala that was not detected by MVPD.

In Experiment 2, standard functional connectivity identified statistical dependence between the FFA seed and other regions of ventral temporal cortex, as well as with early visual cortex (peak MNI coordinates: 12,-90,-6), the right insula (peak MNI: 34,26,1), the thalamus (peak MNI: -9,-23,11), dorsal visual stream area V7 (14, -70, 43) and intraparietal sulcus (IPS, peak MNI: 30,-66,32; Fig 5, in blue, FWE-corrected *p* < 0.05, S3 Table). MVPD additionally identified statistical dependence between the FFA and posterior cingulate (pCing, peak MNI: 8,-46,38), the right superior temporal sulcus (rSTS, peak MNI: 51,-25,-4), the right anterior temporal lobe (rATL, peak MNI: 26 6 -33), right dorsomedial prefrontal cortex (rDMPFC, peak MNI: 8 57 30), and the dorsal visual stream area V3A (peak MNI 15,-88,31; Fig 5, in yellow, FWE-corrected *p* < 0.05, S4 Table). MVPD, unlike standard functional connectivity, did not detect significant statistical dependence between FFA and the amygdala (peak MNI for standard functional connectivity: 22,0,-20). Even after regressing out the global signal and six motion regressors generated by SPM during motion correction (S2 Fig), MVPD detected significant dependence in the posterior cingulate (peak MNI: -2 -39 40), the dorsal visual stream (peak MNI: -29 -76 29; 30 -75 32), occipital cortex (peak MNI: 18 -87 -10; -43 -79 -9).

**Fig 6:**
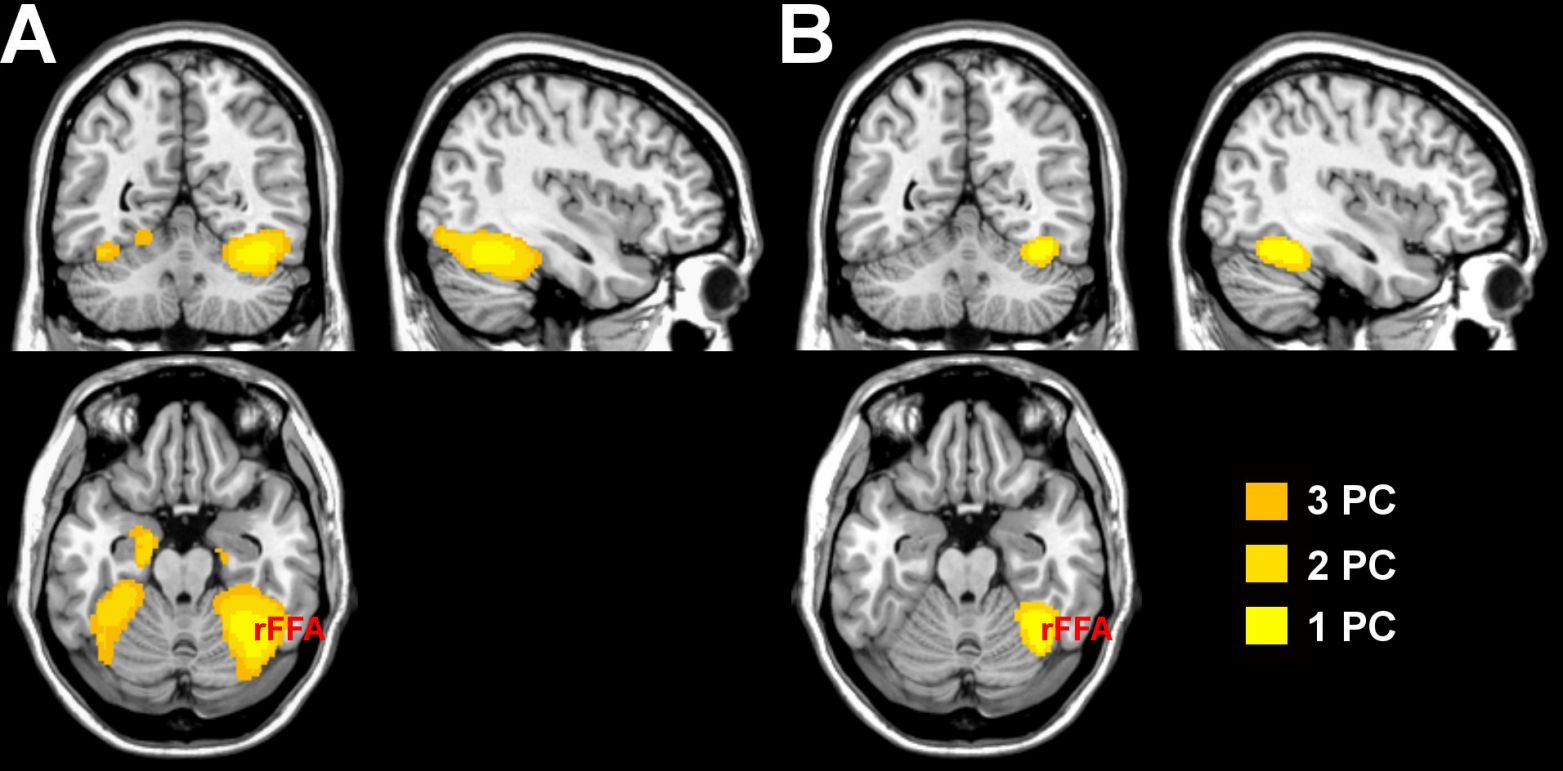
A) Regions with cross-validated R-squared above 5%, as measured by MVPD with 1, 2 and 3 principal components. B) Regions with cross-validated R-squared above 10%, as measured by MVPD with 1, 2 and 3 principal components.

Analysis of voxelwise cross-validated R-squared was performed for mean-based univariate connectivity, and for MVPD with 1, 2, and 3 principal components. Increasing the number of principal components led to a corresponding increase in the voxelwise cross-validated R-squared (Fig 6A for voxels explaining more than 5% of voxelwise variance, (Fig 6B for voxels explaining more than 10% of voxelwise variance). Cross-validated R-squared was also computed after regressing out six motion parameters and the global signal as additional nuisance regressors (S3 Fig). As expected, the greatest voxelwise cross-validated R-squared was observed in the right fusiform gyrus, in the proximity of the seed region’s location. Thanks to the additional contribution of the second and third principal components, variance explained above the 5% threshold was also observed more posteriorly extending towards the occipital face area (OFA), in the left fusiform, and anteriorly extending towards the medial portions of the anterior temporal lobes (ATL). These portions of cortex have been implicated together with FFA in the recognition of faces. [1, 14, 15]. The inclusion of dimensions beyond the first PC improved the modeling of statistical dependence between FFA and other regions implicated in face recognition. The voxelwise cross-validated R-squared with univariate dependence remained below 5% in the whole brain.

Including additional dimensions beyond the first improved our ability to characterize the statistical dependence between responses in the FFA seed and responses in other brain regions that have been implicated in face processing.

As in the case of Experiment 1, we performed an additional analysis removing the univariate signal, obtaining a fully complementary analysis to standard functional connectivity. This analysis revealed multivariate dependence between the FFA and ventral occipital regions despite the univariate signal was removed (S4 Fig).

**Fig 7:**
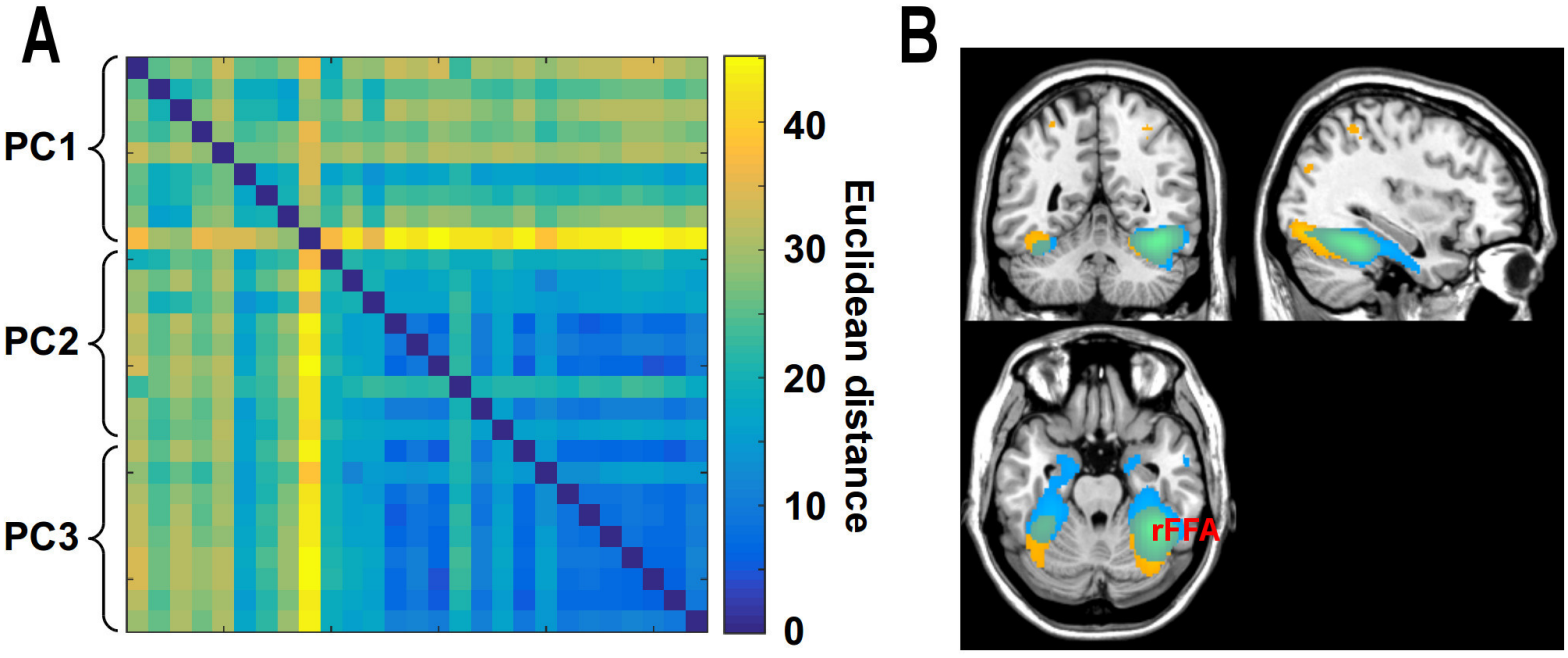
A) Similarity matrix between the whole-brain maps of $\bar{r}$ values obtained with MVPD for each participant reflecting the statistical dependence between each voxel and the first, second, and third PC respectively in the FFA seed. B) Top 5000 voxels showing highest $\bar{r}$ values for the first PC (in yellow), and for the second and third PCs (in blue). Different subspaces of FFA responses show different MVPD profiles, with the first dimension showing greatest statistical dependence with posterior ventral temporal regions and regions in the dorsal visual stream, and the second and third dimensions showing greatest statistical dependence with anterior temporal regions.

We then averaged the MVPD-searchlight maps for the first PC and for the second and third PCs, and we studied the spatial distribution of the top 5000 voxels in the brain showing greatest statistical dependence with the first PC (Fig 7B in yellow) and the top 5000 voxels in the brain showing greatest statistical dependence with the second and third PCs (Fig 7B in blue). The first PC showed greatest statistical dependence with voxels extending posteriorly towards early stages in the visual processing hierarchy, and dorsally towards regions in the dorsal visual stream. By contrast, the second and third PCs showed a different profile: strongest statistical dependence was found with regions extending anteriorly, towards the medial ATL. MVPD revealed different connectivity profiles for different dimensions of FFA’s representational space, individuating two subspaces showing disproportionate statistical dependence with regions involved in early and late visual processing respectively.

## Discussion

This article introduces multivariate pattern connectivity (MVPD), a new method to investigate the multivariate statistical dependence between brain regions. MVPD is characterized by several key properties. First, the BOLD signal in each brain region is modeled as a set of responses along multiple dimensions, with each dimension corresponding to a function of the voxels in that region. Second, MVPD investigates the statistical dependence between two regions by computing the extent to which the responses in the multiple dimensions characterizing one region can predict the responses in the multiple dimensions characterizing the other region over time. Third, with an analogy to MVPA methods, MVPD uses a cross-validation procedure in which independent data are used for training and testing of the models. A subset of the runs are used as a training set to generate parameters which are then tested assessing their ability to predict responses in a left-out independent run. This leave-one-out approach mitigates the impact of noise, improving on most current methods that do not test the extent to which relationships between regions are sufficiently stable to generalize to independent data.

There are two senses in which MVPD is multivariate. First, PCA identifies weighted combinations of multiple voxels that covary over time explaining most of each region’s variance. Therefore, the dimensions that describe the representational space in each region are a combination of multiple dependent variables. Second, in standard functional connectivity, statistical dependence between two regions is measured by correlating two one-dimensional timecourses (the average responses in each of two regions). Instead, in MVPD, statistical dependence is measured by modeling a multiple linear regression that predicts a multi-dimensional timecourse (the responses along the multiple dimensions in one region) as a function of another multi-dimensional timecourse (the responses along the multiple dimensions in the other region).

In the examples described in the present article, dimensions are obtained with PCA as linear combinations of the voxels that tend to be jointly activated or deactivated over time. From a neuroscientific perspective, we can think of each region as consisting of multiple neural populations with selectivities for different properties of the stimuli that have different distributions over the course of the experiment. Each population has different spatial distributions over voxels. This leads different weighted combinations of voxels to having different timecourses of responses, whose dynamics can provide deeper insights into the interactions between regions than the investigation of average responses. Of course, while different populations with different selectivities and different spatial distributions can lead to dimensions with different time courses, it is unlikely that individual dimensions obtained with PCA correspond in a one-to-one relationship to neural populations with a specific selectivity profile. For example, more than one neural population might be collapsed in a single principal component, or populations might not be assigned to dimensions in a one-to-one mapping because of the orthogonality constraints imposed by PCA.

Like standard functional connectivity, MVPD revealed statistical dependence between the FFA and more posterior regions of ventral temporal and occipital cortex, and with regions in early visual cortex. However, MVPD additionally revealed statistical dependence between the FFA and the right ATL, previously implicated in the recognition of face identity [1, 13–15]. Furthermore, MVPD (but not standard functional connectivity) identified statistical dependence between the FFA and the right STS, implicated in the recognition of person identity [21, 28–30] and of facial expressions [31–33]. Standard functional connectivity, but not MVPD, identified statistical dependence between FFA and the amygdala. This can be due to less stable predictive relationships between responses in the amygdala and FFA dimensions beyond the first PC.

Previous studies investigating the functional connectivity of the FFA reported connectivity with the STS in resting state data specifically when the responses in regions selective for other categories were regressed out [34]. MVPD can help to disentangle different kinds of information in the study of statistical dependence: face-specific information might load differentially on different principal components, and the mapping between region can learn to rely specifically on the relevant information. Significant MVPD between FFA and STS might be observed thanks to the potential of the method to rely selectively on a relevant subset of the information encoded in FFA responses.

A recent study investigated effective connectivity between FFA and early visual cortex and STS, including participants with developmental prosopagnosia as well as neurotypical controls [35]. Feedforward connections from EVC to FFA and EVC to pSTS showed reduced strength in DP participants. A promising direction for future research consists in applying MVPD to study differences between patient populations and neurotypical controls, to investigate more closely whether neural differences affect specific subsets of the information encoded within a brain region. In the case of developmental prosopagnosia, MVPD could be used to test whether the reduced connectivity from EVC to FFA and pSTS is specific to particular response dimensions within EVC and FFA.

MVPD led to important improvements in cross-validated R-squared at the voxel level over mean-based univariate connectivity (Fig 6). MVPD using a single principal component already improved variance explained over a mean-based univariate approach. Adding a second and a third PC further improved variance explained in ventral temporal cortex as well as the anterior temporal lobes. In the end, MVPD allowed us to separately investigate the connectivity profiles of different dimensions of FFA’s representational space. In particular, different dimensions showed stronger dependence with posterior and anterior regions respectively. Previous connectivity studies found support for the view that posterior ventral stream regions are an entry node in the face recognition network [36], and previous MVPA studies found evidence of invariant representations of faces in anterior regions [1, 15]. In this context, the present evidence suggests that different FFA dimensions encode information related to FFA’s inputs and outputs respectively.

Future work can investigate the differences in MVPD between different tasks. Whether or not MVPD is sensitive to task differences remains an open question. We consider this a key research direction, in which the greater sensitivity of MVPD can reveal task-dependent changes in the interactions between regions that cannot be detected by standard functional connectivity.

In this study, we showed that MVPD can be sensitive to statistical dependence between regions that is not detected by standard functional connectivity. MVPD has the potential to study in even greater detail how statistical dependence is affected by different tasks. For example, in different tasks, the dependence between two regions could remain similar in overall magnitude, but shift from relying on a particular subset of dimensions to a different subset. MVPD could be used to detect this type of task-dependent changes by analyzing not only the overall amount of variance explained, but the matrix of parameters *B*_*train*_ obtained in different tasks. If some dimensions in one region have a greater influence on responses in another region in one particular task, the parameters in *B*_*train*_ corresponding to those dimensions will increase in that task.

MVPD differs in important respects from previous techniques aimed at studying the dynamic interactions between brain regions in terms of the information they encode. Unlike previous techniques [18, 37], MVPD does not rely on discrimination between categories determined by the experimenter, but on dimensions derived in a data-driven fashion. The data-driven dimensions can be related to properties of the stimuli or the task with a subsequent model (for instance regressing dimensions on conditions, or on stimulus properties using a forward model). Another difference between MVPD and the methods introduced by Coutanche and Thompson-Schill [18, 19] is that the latter characterize each region with a single measure (how well the pattern in a given timepoint can be assigned to one condition or another), while MVPD adopts multiple measures (the values along the multiple dimensions), which can provide a richer characterization of a region’s representation at any given time.

An innovative study [37] investigated the relations between brain regions measuring the correlation between representational dissimilarity matrices in different regions. This approach provides a richer characterization of each region’s representational structure by comparing similarity matrices instead of classification accuracies, but it discards trial-by-trial variability. Furthermore, correlations between dissimilarity matrices can only be computed if the same set of conditions are used to generate the dissimilarity matrices in each region. When the conditions correspond to individual stimuli as in [37] this is not problematic, but if stimulus categories are used it raises the question of whether it is appropriate to characterize the representational spaces of very different brain regions in terms of the dissimilarities between the same set of categories. Taking images of objects as an example of stimuli, categorization based on animacy could be most appropriate for some brain regions, while categorization based on color could be more appropriate for other regions.

An additional approach has used distance correlation to capture multivariate dependences between regions [38], finding more robust results than traditional correlations for inhomogeneous regions. MVPD offers as advantages over this approach the ability to test stability of the dependence between two regions in independent data, and to analyze dependence for different representational subspaces (e.g. Fig 7). This feature of MVPD makes it possible to relate the dimensions characterizing a region’s responses to stimulus properties using forward models, to then investigate what representational content drives statistical dependence between two regions.

More generally, methods to model multivariate statistical dependence can be described by the way in which they model the responses of individual regions, and by the way in which they model the dependence between the regions. Some methods (e.g. [18, 19]) use multivariate methods to generate a unidimensional quantity (e.g. classification accuracy), and measure statistical dependence relating these unidimensional quantities between regions (e.g. with correlation). Other methods (e.g. [37, 39]) map directly the responses along multiple voxels in one region onto responses along multiple voxels in another. MVPD combines the two strategy by initially mapping the multi-voxel responses in each region onto a small set of dimensions (thus reducing the number of parameters that need to be estimated), and then modeling the multivariate relationship between these dimensionality-reduced patterns (e.g. with multiple regression).

By virtue of modeling the statistical dependence between patterns of responses in different regions, which likely correspond to different processing stages, multivariate measures of dependence are related to some extent to the approach of developing computational models of information processing and using them to predict neural responses [40, 41]. Two key differences between these approaches are that at present, computational models of information processing have more sophisticated tools to relate neural responses to stimulus properties, but the model parameters are trained independently of neural responses. By contrast, while multivariate dependence does not yet have the same level of sophistication in linking neural responses to stimulus properties, it gives the neural data a more predominant role in shaping the resulting models, by estimating parameters directly using the fMRI measurements. A recent article [42] built a model of visual cortex more closely inspired to the architecture of the brain, making a step in the direction of combining these two strengths. Future work will be necessary to constrain computational models taking full advantage of the wealth of information available in neural measurements while also tying the neural responses to the stimulus content they represent.

The most important asset of MVPD is probably its flexibility. The framework of 1) modelling representational spaces in individual regions, 2) considering multivariate timecourses as trajectories in these representational spaces, and 3) fitting models predicting the trajectory in the representational space of one region as a function of the trajectory in the representational space in another offers a wealth of possibilities to build increasingly refined models, both in terms of the characterization of representational spaces and in terms of the models of their interactions. For the characterization of representational spaces, in this article we adopted PCA as a simple example, but other methods such as independent component analysis (ICA) and nonlinear dimensionality reduction techniques can also be used. For modelling interactions between regions, we limited the current application to simultaneous, non-directed interactions following an approach similar to functional connectivity, but MVPD makes it possible to model nonlinear maps between representational spaces [43], and to use models that investigate the directionality of interactions using temporal precedence, along the lines of Granger Causality [8], Dynamic Causal Modelling [7], and Dynamic Network Modelling [11].

## Supporting information

S1 Fig Stimuli for Experiment 2. (TIFF)

S2 Fig Functional connectivity (blue) and MVPD (yellow) with the FFA thresholded at FWE *p* < 0.05 with SnPM after regressing out additional nuisance parameters: 6 movement parameters and the global signal. (TIFF)

S3 Fig Voxelwise variance explained by MVPD (thresholded at 10%) after regressing out additional nuisance parameters: 6 movement parameters and the global signal. (TIFF)

S4 Fig MVPD with the FFA after removing both the additional nuisance parameters and the univariate signal, thresholded at FWE *p* < 0.05 with SnPM. (TIFF)

S1 Table Experiment 1: Peaks of functional connectivity with the pSTS seed. (PDF)

S2 Table Experiment 1: Peaks of MVPD with the pSTS seed. (PDF)

S3 Table Experiment 2: Peaks of functional connectivity with the FFA seed. (PDF)

S4 Table Experiment 2: Peaks of MVPD with the FFA seed. (PDF)
