# Supplementary figures and images for: Multivariate pattern dependence
Source: PLoS Comput Biol. 2017 Nov 20;13(11):e1005799. doi: 10.1371/journal.pcbi.1005799 (PMC5714382; doi:10.1371/journal.pcbi.1005799)

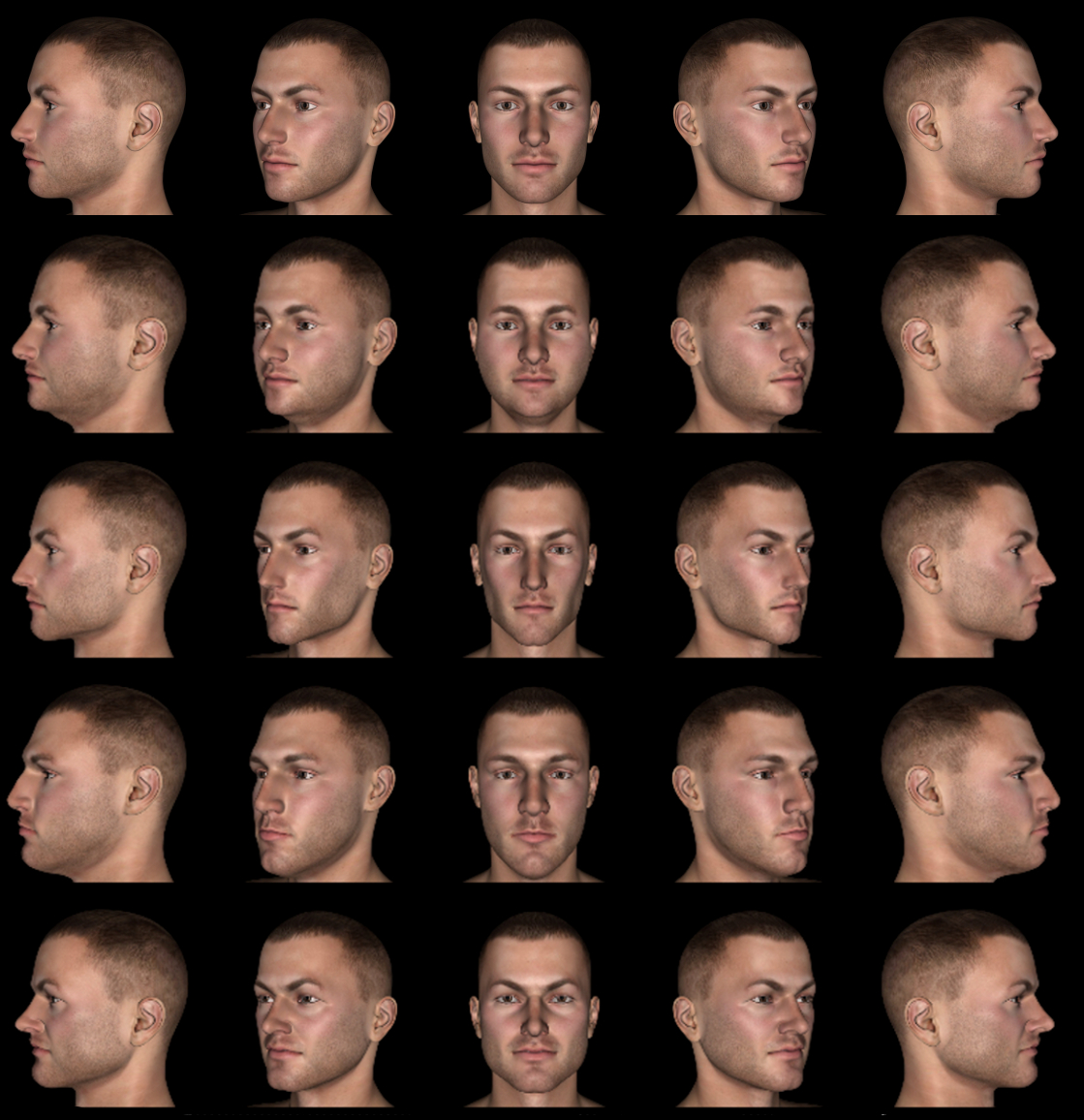

Supplement: S1 Fig — (TIFF) [file pcbi.1005799.s001.tiff]

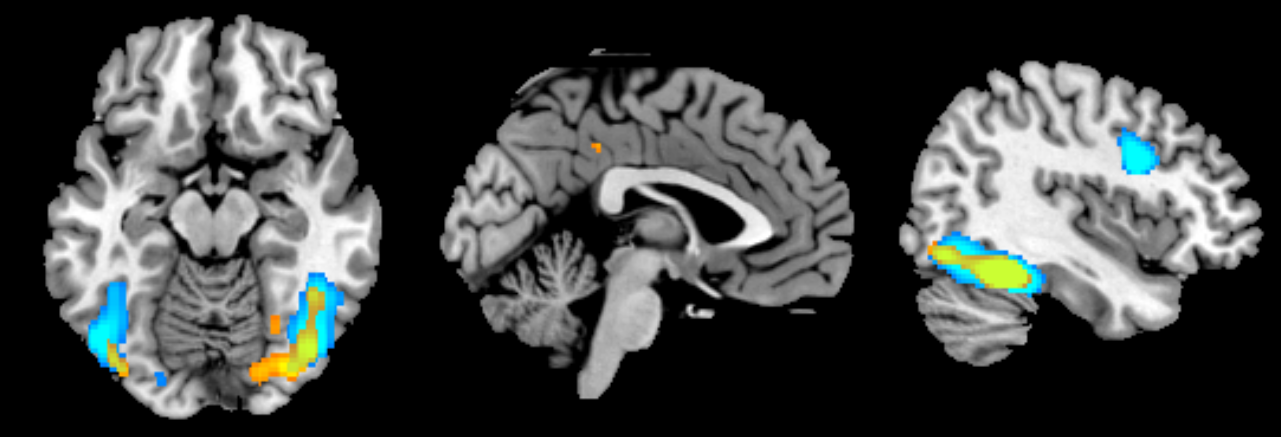

Supplement: S2 Fig — (TIFF) [file pcbi.1005799.s002.tiff]

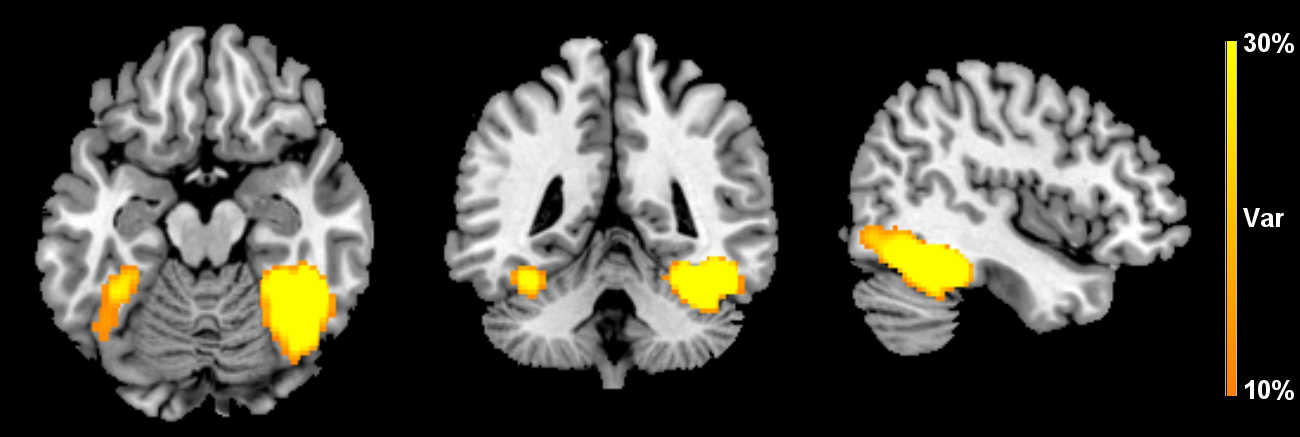

Supplement: S3 Fig — (TIFF) [file pcbi.1005799.s003.tiff]

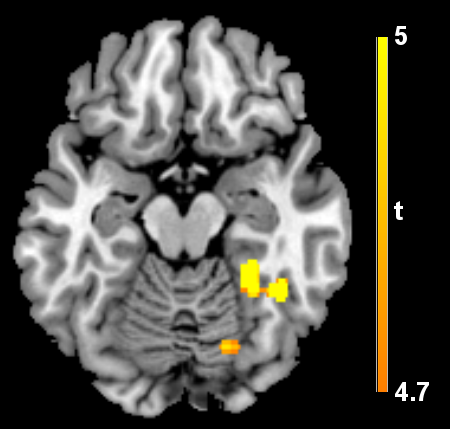

Supplement: S4 Fig — (TIFF) [file pcbi.1005799.s004.tiff]
