# Supplementary material for: Multivariate pattern dependence
Source: PLoS Comput Biol. 2017 Nov 20;13(11):e1005799. doi: 10.1371/journal.pcbi.1005799 (PMC5714382; doi:10.1371/journal.pcbi.1005799)
Supplement: S1 Table — (PDF) [file pcbi.1005799.s005.pdf]

Supplementary Table 1. Experiment 1: peaks of functional connectivity with the pSTS seed.

Region Name	Peak MNI			SnPM T
	x	y	z	
Right STS	54	-9	-15	8.5
Left STS	-52	-27	-6	8.9
Right STS, posterior	55	-57	10	6.1
