# Supplementary material for: Multivariate pattern dependence
Source: PLoS Comput Biol. 2017 Nov 20;13(11):e1005799. doi: 10.1371/journal.pcbi.1005799 (PMC5714382; doi:10.1371/journal.pcbi.1005799)
Supplement: S2 Table — (PDF) [file pcbi.1005799.s006.pdf]

Supplementary Table 2. Experiment 1: peaks of MVPD with the pSTS seed.

Region Name	Peak MNI			SnPM T
	x	y	z	
Right STS	63	-32	-4	7.3
Left STS	-62	-11	-14	7.3
Right STS, posterior	55	-55	13	7.2
Left STS, posterior	-57	-42	1	7.9
Posterior Cingulate	0	-71	34	8.6
