# Supplementary material for: Multivariate pattern dependence
Source: PLoS Comput Biol. 2017 Nov 20;13(11):e1005799. doi: 10.1371/journal.pcbi.1005799 (PMC5714382; doi:10.1371/journal.pcbi.1005799)
Supplement: S3 Table — (PDF) [file pcbi.1005799.s007.pdf]

Supplementary Table 3. Experiment 2: peaks of functional connectivity with the FFA seed.

Region Name	Peak MNI			SnPM T
	x	y	z	
Early visual cortex	12	-90	-7	14.1
Right Insula	34	26	1	13.3
Thalamus	-9	-23	11	9.2
V7	14	-70	43	12.3
Intraparietal Sulcus	30	-66	32	14.5
