# Supplementary material for: Multivariate pattern dependence
Source: PLoS Comput Biol. 2017 Nov 20;13(11):e1005799. doi: 10.1371/journal.pcbi.1005799 (PMC5714382; doi:10.1371/journal.pcbi.1005799)
Supplement: S4 Table — (PDF) [file pcbi.1005799.s008.pdf]

Supplementary Table 4. Experiment 2: peaks of MVPD with the FFA seed.

Region Name	Peak MNI			SnPM T
	x	y	z	
Early visual cortex	12	-87	-12	14.3
Right Insula	51	23	-2	14.7
Thalamus	-9	-23	11	21.1
V7	14	-76	43	16.7
Posterior Cingulate	8	-46	38	13.5
Right STS	51	-25	-4	14.4
Right ATL	26	6	-33	10.5
rDMPFC	53	12	23	16.5
V3A	15	-88	31	13.4
